# Disabled women’s experiences of accessing and utilising maternity services when they are affected by domestic abuse: a critical incident technique study

**DOI:** 10.1186/s12884-015-0616-y

**Published:** 2015-08-20

**Authors:** Caroline Bradbury-Jones, Jenna P. Breckenridge, John Devaney, Thilo Kroll, Anne Lazenbatt, Julie Taylor

**Affiliations:** School of Health and Population Sciences, University of Birmingham, Birmingham, UK; Scottish Improvement Science Collaborating Centre, University of Dundee, Dundee, UK; School of Sociology, Social Policy and Social Work, Queen’s University Belfast, Belfast, UK; Social Dimensions of Health Institute, University of Dundee, Dundee, UK; Child Protection Research Unit, NSPCC/University of Edinburgh, Edinburgh, UK

## Abstract

**Background:**

Women and their babies are entitled to equal access to high quality maternity care. However, when women fit into two or more categories of vulnerability they can face multiple, compound barriers to accessing and utilising services. Disabled women are up to three times more likely to experience domestic abuse than non-disabled women. Domestic abuse may compromise health service access and utilisation and disabled people in general have suboptimal access to healthcare services. Despite this, little is known about the compounding effects of disability and domestic abuse on women’s access to maternity care.

**Methods:**

The aim of the study was to identify how women approach maternity care services, their expectations of services and whether they are able to get the type of care that they need and want. We conducted a qualitative, Critical Incident Technique study in Scotland. Theoretically we drew on Andersen’s model of healthcare use. The model was congruent with our interest in women’s intended/actual use of maternity services and the facilitators and barriers impacting their access to care. Data were generated during 2013 using one-to-one interviews.

**Results:**

Five women took part and collectively reported 45 critical incidents relating to accessing and utilising maternity services. Mapped to the underpinning theoretical framework, our findings show how the four domains of attitudes; knowledge; social norms; and perceived control are important factors shaping maternity care experiences.

**Conclusions:**

Positive staff attitude and empowering women to have control over their own care is crucial in influencing women’s access to and utilisation of maternity healthcare services. Moreover these are cyclical, with the consequences and outcomes of healthcare use becoming part of the enabling or disabling factors affecting future healthcare decisions.Further consideration needs to be given to the development of strategies to access and recruit women in these circumstances. This will provide an opportunity for under-represented and silenced voices to be heard.

## Background

Domestic abuse (domestic violence; intimate partner violence) is the infliction of physical, sexual or mental harm, including coercion or arbitrary deprivation of liberty [1]. A 10-country study on women’s health and domestic abuse reported that 15–71 % of women had experienced physical or sexual violence by their husband or partner [2]. In a 28-country study by the European Union, 22 % of all women in relationships reported physical and/or sexual violence from a current or previous partner since the age of 15 [3]. Domestic abuse occurs in different relationship configurations, irrespective of gender or sexuality [4]. Certain individuals are at higher risk of experiencing domestic abuse and this paper focuses on women with a disability who experience domestic abuse during pregnancy.

### Domestic abuse and disability

Disability is considered a “long-term physical, mental, intellectual or sensory impairments which in interaction with various barriers may hinder their full and effective participation in society on an equal basis with others” [5]. This terminology reflects the social model of disability, recognising that people have impairments but are disabled by social factors [6]. Disabled women are at increased risk of domestic abuse [7, 8]. Over 50 % of disabled women have experienced some form of domestic abuse [3, 6, 9] and they are at particular risk of severe physical violence [10]. Disabled women can face particular forms of impairment related abuse, such as withholding assistive devices [6, 11, 12] or refusing to provide basic care [13, 14]. Domestic abuse among disabled women is linked to difficulty gaining or maintaining employment, maintaining social networks, living independently and mental health problems [12].

### Domestic abuse and pregnancy

An estimated 30 % of domestic abuse begins during the perinatal period [15, 16]. In a European 28-country survey, 20 % of the victims of current partner violence and 42 % of victims of previous partner violence reported physical or sexual abuse during pregnancy. Domestic abuse during pregnancy has a ‘double impact’, potentially harming both the woman and her unborn child. Domestic abuse is a prime cause of maternal deaths during childbirth [17] and pregnant women experiencing violence are at a higher risk of homicide than those who do not experience violence [17, 18]. Domestic abuse is also linked with adverse foetal outcomes, including premature birth, low birth weight, infection, placental abruption, foetal injury and death [19]. Despite these risks, many abused women delay accessing maternity services until the third trimester, placing them at risk of undetected pregnancy complications and inadequate care [20–22].

### Domestic abuse, disability and pregnancy

An estimated 9.4 % of women giving the birth in the UK each year have one or more limiting long standing impairment or health condition [23], and approximately half of these women will experience domestic abuse [24]. According to the World Health Organization, individuals who fit two or more vulnerability categories (women, children, disabled people, migrants, people with HIV/AIDs or experiencing domestic abuse) face particularly complex barriers to healthcare [1]. Research in the UK [6, 25, 26] and US [12] has provided some insight into disability and domestic abuse more generally, however, there is a lack of research about access to services for disabled women who experience domestic abuse during pregnancy. Potentially, the co-existence of disability and domestic abuse may compound barriers to accessing good maternity care [6, 7, 9, 27]. Until there is a good understanding of the relationship between disability, domestic abuse and pregnancy, the best strategies for achieving universal access to maternity care will remain elusive. The purpose of this study was to address this gap in empirical knowledge by listening to disabled women’s experiences of accessing and using maternity services when they were affected by domestic abuse.

### Research questions

For disabled women who experience domestic abuse, what are the:

1. Barriers and facilitators to access and utilisation of maternity services?
2. Impact of previous experiences of maternity services on future access and utilisation?
3. Implications of the barriers, facilitators and experiences for service delivery and improvement?

### Theoretical framework

**Fig. 1:**
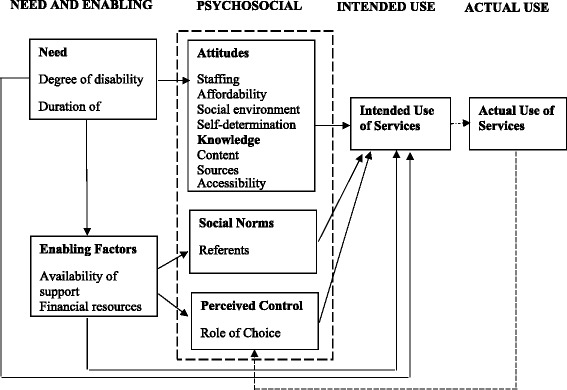
Bradley and colleagues’ (2002) revised version of the Andersen Model

Clear and consistent use of theory improves the quality of qualitative research [28]. The theory underpinning this study was Andersen’s model of healthcare use [29], which presents healthcare use as determined by people’s predisposition to use services, their need for healthcare and the enabling and disabling factors that influence their access to care [30]. Originally developed in the 1960s to explain and predict the factors influencing the use of acute services, the Andersen model has been subject to various modifications and revisions since its inception [29]. From our extensive review of the literature undertaken for an earlier stage of this study [22] we believe we are the first to explore its use in the context of maternity care. Bradley and colleagues [31] criticised the model for ignoring the psychological factors influencing healthcare use and, in collaboration with Andersen, added an additional four components to the model: attitudes; knowledge; social norms; perceived control [31]. These additional components combine the strengths of models such as the Theory of Planned Behaviour, which focus on individual factors, with the structural focus of the Andersen model [32]. We anticipated that individual factors, as well as aspects of the environment, would impact upon the health-seeking behaviours of disabled women affected by domestic abuse, therefore we applied Bradley’s revised version of the model to our research (Fig. 1).

## Methods

We conducted a qualitative Critical Incident Technique (CIT) study in Scotland. CIT was developed to evaluate and improve the outcome of flying missions during World War II [33] and has since been embraced by different disciplines. Data collection and analysis in CIT focuses on actual behaviour and ensures that research is based on how things really are, rather than individuals’ perceptions of how things should be [34]. This is especially important in applied healthcare research. Also, CIT’s potential to uncover the specific behaviours, motivators and consequences behind actual healthcare access and utilisation means that it was aptly suited to our study. We wanted to identify how women approached maternity care (behaviour), why they accessed services (motivators) and whether they were able to get the type of care that they needed and wanted (consequences). CIT can be used in conjunction with any theoretical framework to classify, understand and interpret participants’ behaviours [33]. Focusing on behaviour, motivators and consequences complemented the main domains of the Andersen model [31] and gave us a practical means of identifying actual examples relating to need and enabling factors; psychosocial factors; intended use; and actual use.

### Recruitment

We recruited women who: had seen a health professional in relation to pregnancy; had experienced domestic abuse; and lived with a health condition or impairment (physical, mental health, sensory or intellectual). Our primary recruitment strategy involved identifying and approaching potential participants through Scottish Women’s Aid. We also targeted other charitable organisations and publicised the study across a variety of parenting, disability, and domestic abuse charities in the UK; via our Facebook page (www.facebook.com/maternitystudy); in a local newspaper and two local radio stations. Studies like ours require a multi-faceted recruitment strategy because participants may be hidden or hard to reach and the sensitive nature of the topic may deter people from participating because of fears around safety, comfort and disclosure [34]. Specifically, women who have experienced domestic abuse are concerned about fear of reprisal and stigma, which may hamper their willingness to participate. All these issues may account partially for our challenge in recruiting women to the study [22].

### Participants

Five women participated in the study. We collected limited demographic information to protect women’s anonymity. All women had more than one child and had experienced domestic abuse during at least one pregnancy. Women’s disability status is summarised in Table 1.

**Table 1:** Participants’ disability status

Hanna	Congenital physical impairment & mild learning difficulty
Jessica	Congenital physical impairment
Rachel	Long term mental health condition
Kirsty	Long term mental health condition
Laura	Long term mental health condition & acquired physical impairment

We identified a further nine women that met our inclusion criteria, however, they were either unable or unwilling to take part. This reflects the complexity of recruitment and is an important finding in itself. Our recruitment to this phase fell 50 % short of our anticipated sample. Knowing that there are women in this situation who remain under-represented in the literature reinforces the importance of continuing to research difficult topics despite recruitment challenges. Although sample size in qualitative research is not an important indicator of quality or rigour [35] we recognise the impact of our sample size on transferability of findings. We argue however, that the strong theoretical base of our study, coupled with extremely limited knowledge in this important area make the findings of interest, relevance and usability.

### Data generation

We conducted individual CIT interviews with women to elicit their experiences of accessing and utilising maternity care. In accordance with CIT, we asked participants to describe specific encounters with health professionals throughout their pregnancy journey [36]. They were encouraged to elaborate on the barriers they faced when accessing care and how they overcame these. We used prompt questions (Table 2) that corresponded to the key elements of CIT [32] and the theoretical framework. We wanted to elicit uninterrupted, self-directed critical incident narratives so we followed this flexibly. Interviews were carried out face to face (*n* = 3), via Skype (*n* = 1) and via e-mail (*n* = 1). We offered a range of methods to make it convenient for women to participate.

**Table 2:** Interview prompt questions, aligned with the theoretical framework and methodology

Prompt questions	Andersen’s model	CIT
Tell me about a situation where you needed to see a health professional about your pregnancy or post-natal health	Actual Use	Behaviour
● What happened?		
● Who did you see?		
Why did you need to see them?	Need Intended Use	Motivators
Did you get the care you needed?	Actual Use	Consequences
● In relation to pregnancy?		
● disability issues?		
● domestic abuse?		
What helped/or would have helped you to get the care you needed?	Enabling Factors	Context
What made it difficult to get the care you needed?	Disabling Factors	Context
● Did maternity services accommodate your disability needs?		
● How did domestic abuse affect your access to care?		
How did that affect you? How did it affect your child (baby)?		Consequences
In what ways did using maternity services make a difference to you? OR How would better maternity care have made a difference to you?		Consequences

### Data analysis

CIT is concerned with the amount of incidents, rather than the number of participants [36]. Although we only interviewed five women, they generated a total of 45 complete critical incidents. In CIT, an ‘incident’ is identifiable in the data when an experience is described in detail, including the contextual and antecedent factors leading up to the experience and the consequences arising from it [37]. To enhance reliability, two researchers (*initials*) analysed the interview transcripts to identify and count the critical incidents. Using Ritchie and Spencer’s [38] framework approach to analysing qualitative data, both researchers conducted an inductive thematic analysis of the interview data independently. JB had undertaken all the interviews, so a dual approach to analysis provided a reflexive means of checking the ways that participant responses had been shaped by the interview process. In line with the framework approach, our analysis involved the process of sifting, charting and sorting the data into key themes. These were subsequently discussed and agreed within the rest of the team and the critical incidents and emerging themes were mapped to the theoretical framework.

### Ethics

Ethical approval was granted by the University of Dundee Research Ethics Committee (reference: UREC 12116)]. Informed consent was obtained from all women who participated in the study. Domestic abuse research carries specific ethical challenges around ensuring participant safety and anonymity [39]. All interviews were conducted in participants’ preferred location and mode. Individual and Helpline supports were in place throughout. Although no woman disclosed on-going abuse or current risk, had they done so we would have had a duty of care to protect her and any children. Lines of referral and safety planning would have been explored as a matter of priority. None of the women demonstrated visible signs of distress. All stated that they were pleased to take part and wanted their experiences to help improve services for other women. Their names have been changed to protect their anonymity. The research team supported each other throughout the study to address any issues of vicarious trauma and to facilitate reflexivity, making sure that we were attentive to the voice of participants rather than allowing our own perspectives to dominate.

## Results

We used Andersen’s model to organise our inductively derived themes and critical incidents. There was considerable fit between our data and the four psychosocial domains: 1) knowledge; 2) attitudes; 3) social norms; 4) perceived control [31]. Theoretical frameworks are often adapted to achieve best fit with the data [28] and, although we gave credence to the other components of the model, we primarily organised our findings around these domains. Critical incidents typically mapped to one predominant psychosocial domain, but could also have relevance to others (demonstrated in parentheses throughout the findings section). Table 3 summarises the distribution of the selected critical incidents across each of the four domains, as well as the distribution of incidents across the entire dataset. We cannot draw statistical conclusions from this, however it highlights the salience of certain factors in relation to others, particularly the importance of perceived control.

**Table 3:** Distribution of critical incidents across the four psychosocial domains

Psychosocial aspects of the Andersen model within selected critical incidents				
	Attitudes	Knowledge	Social Norms	Perceived control
Facilitators	3	1	6	6
Barriers	2	1	4	6
Total	5	2	10	12
Psychosocial aspects of the Andersen model across all critical incidents (*n* = 45)				
	Attitudes	Knowledge	Social Norms	Perceived control
Facilitators				
Barriers				
Total	19	17	13	31

### Having knowledge

When individuals are fully informed and know what to expect from their healthcare experience, they are more likely to use services [31]. For the women in our study, knowledge was empowering. It enabled them to make their own choices and feel in control of their care decisions:

I found the information because I sought it out. With my first two births, I didn’t know better… there’s enough patient education resources available where you can make decisions for yourself… When you’re empowered with the information, just if you’re given the information about the risks and benefits or informed consent about things, it kind of gives you your power back. And being from a disabled situation or an abuse situation, it’s kind of the power that you’ve lost that you never knew that you lost until you get it back (Knowledge; Control) (Laura)

For knowledge to be empowering, women needed the right information (content) at the right time (amount) in the right way (accessibility). For disabled women, information-giving and access to care must be tailored to individual need. For example, Kirsty’s memory difficulties meant that she struggled to remember information, resulting in missed appointments and inadequate maternity care utilisation:

So I’ve not heard from anybody in weeks. And then I found out I was supposed to make my own appointments, I was like, “Oh, great.”…so I was like, “Really?” So I didn’t even know I was to do that, I’d forgotten…until right later on in the pregnancy, I think it was more like 30 weeks or something. (Knowledge) (Kirsty)

Use of medical terminology was also confusing and unfamiliar jargon made it difficult for women to feel fully informed:

Because I’m not so mobile, they gave me an injection to thin my blood out, which was called [pause] Cycli – no. I’m trying to think. Cyclizy or something, something along those lines… listening to all this medical jargon. They say something and you’re like that, “What? What is that?” (Knowledge) (Hannah)

Biomedical jargon excludes women from fully accessing information about their care, particularly for women with a health condition or impairment that affects their ability to process information. It is notable that Hannah, who had a learning difficulty, found the use of jargon most frustrating. Informed decision making is fundamental to effective birth planning and the empowerment of women in childbirth [40]. National Institute for Health and Care Excellence (NICE) guidelines [41] offer best practice advice on the care of women in labour and the principles of good care can be extrapolated to other maternity contexts. Akin to our findings, the guidelines highlight the importance of accessible information that takes into account any additional needs of women such as physical or cognitive disabilities. They also reinforce the need to promote good communication and shared decision making between women and healthcare professionals.

We identified several incidents where women reported anxiety and panic because staff did not provide adequate information or dismissed women’s concerns:

When they were doing the section, there were voices being thrown back and forth saying, “No, give her a spinal,” and then it was like that, “No, give her a general,” and they were like that, “No, hold on, she’s going to go for a spinal,” and they were like that, “No, she’s got to go for a general.” I just didn’t know what was happening (Knowledge; Control) (Hannah)

Lack of information or incorrect information diminished women’s trust in health professionals:

I phoned the doctor and said… “I think I’ve got an iron deficiency,” because I’d had blood tests done, and my iron one week I’d had it done it was 11.4, and then it was 10.1, and I was just like totally exhausted. And he was like, “No, that’s normal, you’re fine,” and I was like, “Alright.” So I went on a few more days, and it was just getting worse. I phoned the midwife and she was like, “No, that’s completely wrong.” She got the iron tablets issued for me. Once they’d kicked in, I was a lot better… That was a bad experience I had with that, it was just like a nightmare… (Knowledge; Attitudes; Control) (Kirsty)

This confirms Bradley and colleagues’ [31] suggestion that knowledge and attitude are closely linked; when women in our study were informed and had confidence in their healthcare provider, they appeared to be more likely to have positive attitudes towards using future services. Conversely, lack of knowledge deterred them from using services, for example: “I just don’t want to see another midwife, I don’t want to see another doctor, I don’t want to see another obstetrician” (Hannah). Optimal access to maternity care for disabled women experiencing domestic abuse therefore requires that women are given enough information to feel empowered and able to trust service providers.

### Women’s attitudes towards health professionals

Personal views about health services and care providers have direct impact on intended use of services [31]. In our study, women’s attitudes towards using maternity services were typically determined by past experiences. Negative past experiences could deter future maternity care access and utilisation:

[When I was in labour with my second baby] I wanted to get down and walk to the loo [toilet], but I found it difficult to get down because the bed was so high… [The midwife] brought this bedpan, but I just couldn’t get on [it] between contractions… She got the bedpan and actually threw it across the room, said that I was wasting her time… I was really scared to then have my third [baby], because I thought, “Oh, is the care going to be the same?” I don’t want somebody treating me like that (Attitudes; Control) (Rachel)

With the other two [pregnancies]… I went to the doctor straight away. It wasn’t like that [this time]… I didn’t want to go through, you know, it’s almost traumatic just to go to the doctor, because you have these strangers, they’re very invasive with pelvic exams, and doing a bunch of procedures without even asking your permission… (Attitudes; Control) (Laura)

On the most part, women approached services tentatively for fear of judgement from health professionals:

I thought the midwives were going to be stuck up and right up themselves. But they weren’t. I even said that to my consultant, I was like that, “Are the midwives up themselves? Are they stuck up?” and she was like that, “No, they’re really, really nice”… So I was like, “Right, okay then.” And I got admitted, and they were. They were exactly how [she said they would be], if not more (Attitudes) (Hannah)

Hannah’s experience is echoed in other studies with disabled women experiencing domestic abuse, suggesting that anticipation of poor relationships with health professionals is a critical barrier to accessing care [42, 43]. Hannah’s example demonstrates how women’s attitudes can be transformed by positive interactions that defy initial fears and expectations. It is therefore essential that every practitioner-patient interaction is positive and non-judgmental, taking all available opportunities to change women’s negative perceptions of maternity care which are often a barrier to utilising services [22].

### Social norms

Social norm refers to the culturally determined standards that shape how service users and healthcare providers interact [31]. Social norms - women’s perceptions of what other people think of them – have a significant impact on the decision to access maternity care. The biggest concern for women in our study was the desire to be seen as “normal pregnant women”. They were concerned that common societal misconceptions about disability and domestic abuse would affect the ways in which health professionals treated them:

Some of the forms [in my previous pregnancies], I didn’t really fill much out. And I don’t know whether I did put that much in detail, I don’t know, it’s a bit embarrassing to put things down… just because I’ve got that diagnosis, I’m not mental or anything. I am normal… but I think just because of everything that’s happened this time, like my husband [being more] abusive towards me [there is] more in my record this time. Because this is the first pregnancy that … a midwife that deals with that kind of thing was asked to come on board and offer me a bit more support (Social Norms) (Rachel)

I was a little bit reluctant to share my history and everything that I knew was relevant, but at the same time I didn’t want to open myself up… They ask you questions in their questionnaire, “Have you been involved in domestic abuse? Have you done this and this and this?” and it’s kind of like a piece of paper, and you check off all the problems that you have with yourself… why do they even want to know all this stuff? And I really feel – and I might be just jaded or cynical about it, but I really do feel it’s because they want to judge you about what kind of decisions you can make for yourself. (Attitudes; Social Norms) (Laura).

Women recognised that they might often need specialist support, but they did not want to be perceived by staff and other service users to be different or incapable as mothers. They wanted to receive the same treatment as all other pregnant women. This included being given the same choices as non-disabled women without domestic abuse experiences. Within mainstream midwifery, there has been a gradual shift towards woman-centred care in which women are involved as active partners in decision making and their preferences are respected [44, 45]^.^ This has challenged the deep rooted social norms within healthcare settings that traditionally established health professionals as ‘experts’ and service users as passive recipients of care [46].

For our participants, it was important that health professionals respected them as experts in their own care. All five women had more than one child and, while they wanted maternity care practitioners with sound technical expertise, they also brought their own knowledge and experience from past pregnancies. Unfortunately, these aspirations were rarely actualised. Because their pregnancies were perceived be health professionals to be “abnormal”, their care was dominated by the social norms of a traditional medical model, rather than those of woman-centred care. We identified ten incidents where women’s choices were denied; their preferences ignored and their sense of agency compromised. As illustrated in Rachel’s and Laura’s experiences, women’s experiential knowledge of pregnancy was frequently dismissed by health professionals:

[I was in labour] and I felt like the contractions were coming and it was getting quite sore. Then I asked for gas and air, but they were like, “No, you can’t have that,”… [but] I felt I did need that gas and air, because that’s what I do to help me cope (Control; Social Norms) (Rachel)

I was sent to the high-risk clinic… and I printed out a whole sheet of all the memories I could have from my previous birth, so my health history and everything like that. I spent a lot of time working on this, and they were just like, “Oh, okay,” and tossed it to the assistant. And every time I would go they would ask me the same questions and I’m like, “Okay, I’ve already answered all this for you guys, I took the time to print it out,” but then it was like they didn’t even read my medical history (Control; Knowledge; Social Norms) (Laura).

Failure to listen to or respect women’s voices undermined their sense of control. When maternity care professionals valued women’s knowledge and opinions as inferior to their own, this reinforced stereotypical ‘passive patient’ and ‘expert practitioner’ norms. Our findings reflect broader midwifery research that highlights women’s lack of involvement in care decisions. For example, a survey of 3,542 women in Australia [47] found a lack of informed decision making across a range of procedures.

Ultimately, childbirth is neither ‘normal’ nor ‘abnormal’. It is, quite simply, a childbirth journey - a uniquely individual experience. However, whenever women are allocated a risk label there may be an accompanying anticipation of pathology. In turn, care is likely to be organised around the ‘risk factor’ and individuality merges into the background. Although the following quotation is not a complete critical incident, we have included it here because it captures this dilemma so well. Talking about her referral to the ‘high risk’ clinic, Laura says:

It’s really easy to put a stereotype on someone, like “Oh, well, this person cannot make good decisions for themselves because a) they’re disabled or b) they made such horrible decisions to put themselves in an abusive situation,” which the two don’t have anything to do with each other a lot of times. I think it’s understood in society that if a woman is in an abusive situation, she can’t make good decisions for herself (Laura)

The only way to ensure that care remains specific to the individual woman is to encompass the foundations of woman-centred care [40]: working with women as partners; respecting their expertise; and making decisions based upon individuals rather than stereotypes or entrenched professional norms. All these things situate women in a context of control rather than disempowerment and challenge the prevailing social perceptions that disabled women who experience domestic abuse are less able than “normal” women to make informed choices about their own healthcare.

### Perceived control

Perceived control is the degree to which people are able to have a say in what happens to them [31]. For the women in our study, perceived control emerged as the most important factor shaping their maternity care experiences. All five women raised the issue of control and 31 out of 45 incidents related to women’s attempts to gain control. Women’s ability to make informed choices featured repeatedly in our analysis and this strong theme of ‘control’ concurs with others’ research [47, 48]. A qualitative study with 101 parous women in the US highlighted an endemic lack of control for women, with 46 % spontaneously mentioned the term ‘control’ in interviews [47].

The women in our study sought to control when, how and to whom they disclosed their pregnancies, disabilities or domestic abuse experiences. Disclosure is a well-recognised concern for both disabled people [49] and victims of domestic abuse [50–52] and the women in our study worried about losing control over their bodies, their family and their baby following disclosure. However, there often came a point where contacting maternity services was seen as necessary and the desire to protect their unborn baby outweighed personal concerns about accessing care. To illustrate, Jessica - who faced increasing physical abuse during pregnancy - was willing to risk her worst fear in order to get reassurance that her baby was safe and healthy:

Jessica: I was scared of social services taking my baby so I waited until I was five months pregnant before I had a scan

Interviewer: So what made you go for the scan at five months?

Jessica: To check the baby’s health and due date (Control) (Jessica)

Ultimately, all women saw maternity care utilisation as a necessity, regardless of personal fears and anxieties. This reflects Andersen’s [29] position that pregnancy is a “biological imperative” for maternity care utilisation (p.3). Women’s decision to make initial contact with services was therefore not a case of “if” but “when”. To gain a greater sense of control, all women sought out an ally that they could trust with their information:

I was quite open with the midwife, and I just said… that the way I was treated with my daughter was totally unfair. I was treated like a criminal. I’d had a breakdown. They were supposed to be there to help, but all they did was take my daughter away from me, and treated me like…it was as though I had committed a murder. That’s the way I was being treated… So, I did say to the midwife, “I’m not going to get care if I know that you’re going to disclose information [without my permission].” I said, “I need your support.” (Attitude; Control) (Rachel)

Contacting services marked the beginning of a healthcare journey that could be physically and emotionally uncomfortable. Whilst these concerns are relevant to all pregnant women, anxieties were heightened for women with a history of sexual abuse or for women who found it difficult or painful to tolerate examinations because of limited physical mobility:

I did tell them [maternity staff], “I cannot lie flat on my back. I sit up in a chair.” Because the first [midwife] came in when they were hooking me up and they put the bed in the lithotomy position or however you call it, and I said, “I cannot lie like that,” and they said, “Oh, well, you have to for this exam,” or, “You have to.” And when I went to the [other] midwife, she said, “Okay, well let me get a couple of pillows and put it behind you,” which made all the difference in the world (Control) (Laura)

Indeed, although many of the issues raised by the women in our study could apply to women without an impairment and history of abuse, it is notable that all our participants were clearly focused on the issue of control. As disabled women, they had experienced social exclusion and limited participation in daily social activities. Moreover, they had experienced having their freedoms removed by an oppressive partner. This fuelled their determination to exercise their independence and have a say in what happened to them, including making choices about their access and utilisation of maternity care.

## Discussion

Very few studies have been able to explicate the complex inter-relationship between disability and domestic abuse during pregnancy. Typically, studies have either focussed predominantly on one or the other [22]. Moreover, the studies which have identified barriers to maternity care for women in this situation tend to focus on the effects of disability – most notably environmental inaccessibility - rather than domestic abuse [22]. Physical accessibility was not raised by the women in our study as a particularly significant barrier to accessing care, with the exception of two women who reported difficulties during examinations because of limited physical mobility. Instead, women’s narratives were dominated by fear of disclosure, and the resulting consequences of staff judgement and loss of control. Unlike non-disabled women, disabled women’s pregnancies were perceived as “abnormal” and high risk even without the presence of domestic abuse. Women therefore experienced a double fear of disclosure; they risked being judged on the basis of both disability and domestic abuse. Other studies have shown that disabled women are often stereotyped as unfit mothers [53], and this is further compounded by the impact of domestic abuse.

Enquiring about domestic abuse is an important step towards providing appropriate support to women [54]. However, our findings show that disclosure is not only hampered by women’s fear of disclosing but also the lack of accessible and inclusive information that takes account of women’s additional needs. While we cannot make any definitive correlations between disability and domestic abuse, our findings add to existing literature which highlights the potentially compounding effect of disability and domestic abuse on women’s access to good, equitable maternity care. It provides a foundation for future work in this area and more research is required to understand the unique experiences of disabled women affected by domestic abuse during pregnancy.

### Limitations

The study was limited by the small number of participants and we have already discussed the implications of this for transferability. Because recruitment was a challenge, we had to adopt different mechanisms to involve women which included different media, including Skype and email. Some may view this approach as unacceptably ad-hoc. We accept that employing multiple data collection modes may hamper the reliability of some studies. It is also possible that the use of different data collection methods, particularly email, may have compromised the consistency and depth of the data generated. But we view our strategy as a reflexive means of reaching women and attempting to include their perspectives. We believe strongly in the importance of inclusive research design and using these approaches ensured that we captured the voices of women who were unable to meet face to face due to disability or domestic abuse. The richness of our data and large number of critical incidents collected means that we have been able to identify some key challenges in the access of maternity care services for this population. This makes the study meaningful in its own right. But additionally, the findings have been crucial in informing the next phase of the research (where maternity staff have developed strategies to overcome the issues identified by women) (*forthcoming publication*). Our study has highlighted that future research is required in relation to provider-focused strategies that may be effective in reaching out and caring for women who potentially experience compound marginalisation as a result of their disability and domestic abuse.

## Conclusion

Equal access to good maternity care is essential to the health and wellbeing of all mothers and their babies. [55] It is imperative that particular groups in society are not excluded from healthcare provision on the basisof biological, socio-economic factors or discrimination [56, 57]. Our study has highlighted some important issues for disabled women affected by domestic abuse when accessing and using maternity services.Crucially, we have demonstrated that the factors influencing access and utilisation of healthcare services are cyclical. The consequences and outcomes of healthcare use (women’s satisfaction with the service they received) in turn become part of the enabling or disabling factors affecting future healthcare decisions.Corresponding to the work of Piotrowski and Snell [58] our findings suggest that the more positive women’s experiences, the more likely they are to make contact with health services again. Conversely, poor experiences lower women’s expectations of services making them less likely to use health services or place their trust in health professionals [59]. Our findings suggest that women’s attitudes to accessing care can be transformed by positive experiences. It is essential that maternity care providers empower women throughnon-judgemental, supportive attitudes, allowing them to exercise control in relation to access and utilisation of maternity services.

We have begun to address a gap in knowledge regarding maternity services for disabled women whoexperience domestic abuse [6, 7]. Clearly future research is required in this area. To further validate the findings a useful comparison in a future study is suggested between four groups of women: 1. Women with adisability and domestic violence; 2. Women with a disability without domestic violence; 3. Women without a disability and domestic violence; and 4. Women without a disability and without domestic violence.Additionally, studies on provider-focused strategies to improve access to maternity care are needed, particularly those that explore how to reach out and care for women who potentially experience compound marginalisation as a result of their disability and domestic abuse.
